# Health and disease among Somali primary school children in Hargeisa

**DOI:** 10.1080/16549716.2019.1598648

**Published:** 2019-04-23

**Authors:** Magnus Andreas Nordstrand, Daniel Stenberg Saxe, Mohammed Abdirizak Mohammed, Mary B. Adam

**Affiliations:** a Medical Department, Edna Adan University Hospital, Hargeisa, Somaliland; b Maternal Newborn Community, AIC Kijabe Hospital, Kijabe, Kenya

**Keywords:** School health screening, Somali children, malnourishment, impaired vision children, vaccination status

## Abstract

**Background and objective**: Limited data exist on health conditions of school children in Somaliland. School Health Intervention Pilot Program (SHIPP) was conducted through Edna Adan University Hospital to screen children and offer interventions. We present the results of the general health screening of the school children, and also describe the association between nutritional status and other variables.

**Methods**: In this cross-sectional study children from two public primary schools in Hargeisa were assessed for general health by nursing students. Nutritional status was assessed by BMI-for-age z-scores and visual acuity by Paediatric Snellen Chart.

**Results**: We screened 2,093 children aged 4–19 years; 58% were boys. Very low BMI-for-age (z-score < −3) was detected in 10%; 6% had visual acuity below 0.7; 26% had dental caries. Children reported low exposure to health services: 33% reported no prior vaccination; 46% reported they had never visited a health clinic or hospital.

**Conclusion**: A significant number of children were malnourished, had reduced visual acuity or treatable infections which could impact their ability to learn. Public schools are a feasible entry point for public health action including screening, treatment, and referral in fragile countries.

## Background

Schools and school systems present a tremendous opportunity to reach children in need of health care services. School-based health screenings identify children with treatable conditions that impact their ability to learn as well as presenting opportunities for broad-based health education and community engagement.

In war torn areas where basic services, including health services, have been destroyed, school health programs have a unique potential to contribute to child health and well-being [1]. Somaliland, located on the horn of Africa, is still recovering from a long civil war that ended in 1991 and resulted in the destruction of more than 95% of its cities with the death of more than a quarter of a million people [2]. Educational opportunities and health services are gradually improving through collaborations between government agencies, public and private organizations, universities, and hospitals [1]. This growth in educational services in Somaliland presents an opportunity for public health action such as screening, referral, and treatment of conditions which can impact learning. In addition, it presents opportunity for health education and community engagement.

Primary education is strongly encouraged but not compulsory in Somaliland. The educational system includes primary (grades 1–8) and secondary (grades 9–12) schools. The schools are both public and private, including integrated faith-based Quranic schools [3]. Out of the 1,145 schools registered in 2016, 86% were public [4]. Yet as of 2015, only 46% of the primary school aged children in urban areas and only 20% in rural areas were enrolled in school [3]. Overall enrolment is low with only 33% of the boys and 29% of the girls aged 5–12 years, attending any educational facility [5], but girls (46% of enrolled students) are almost as likely as boys to attend school [4]. Public schools charge fees of between $1 and $10/month; reducing accessibility for the poorest children. School entry is recommended at 6 years, but 71% start a later age [3]. Reasons include economics and nomadic life styles with families moving in and out of areas with schooling opportunities.

Data on the health status of school age children in Somaliland is extremely limited. Minimal data on children under five is available. A 2009 National Micronutrient and Anthropometric Nutrition Survey reported that among children aged 6 to 59 months, 10% had moderate malnutrition, (weight-for-height, wasting, z-score between −2SD and −3SD), and 4% were severely malnourished (z-score < −3SD) [6]. This can be compared with figures from a meta-analysis of children under 5 years in East Africa which showed that the proportion of wasting (weight-for-height z-score < −2SD) was 9% in Ethiopia, 6% in Burundi and 4% in Kenya [7]. Current development goals include reduction in the prevalence of wasting among children under 5 years to less than 10% by 2021 [8].

The Somaliland Multiple Indicator Cluster Survey 2011 examined child health reporting 43% of children had not received any vaccinations and only 7% of the children (12–23 months) were fully vaccinated (Expanded Programme on Immunization). In children under 5 years of age, 13% of had diarrhoeal disease one week prior to the survey; 6% reported symptoms of pneumonia during the two weeks preceding the survey and only 31% of the reported conditions were taken to an appropriate health provider [9].

The present study is based on a School Health Intervention Pilot Program (SHIPP) which was implemented in two public primary schools in Hargeisa. Our specific objective was to pilot a school health screening program exposing trainees to this public health opportunity and to describe health status of these school age children in Hargeisa.

## Methods

The two schools, Fadumo Bixi and Maalin Daud, are primary schools serving approximately 2,320 children in grades 1–8. These schools were selected because they were located in the catchment area of Edna Adan University Hospital (EAUH), a partnering organization that runs training programs in nursing and public health. The program design, based on review of published work on school-based screening, was adapted to be feasible in our context [10–13].

Baseline assessment demonstrated that neither school had drinkable water or even running water [14]. All water on the school compound was brought in via water trucks. The schools had approximately 1 toilet/100 pupils, far less than WHO guidelines (1:20–40) [14].

Nursing students, from the Nursing and Public Health School at EAUH, received theoretical and practical training on screening of children in a one-day training seminar that was conducted by three medical doctors at EAUH. One hundred nursing students participated. Five medical doctors and two licensed nurses participated to supervise students. These students and supervisors were divided into 10 teams. The screening was implemented over 5-day cycles in each school in 2017. Screening included demographic and current health information. Children underwent a general physical examination by student nurses which included weight, length, visual acuity, and inspection for BCG scar. Children responded to questions about their general health, immunization status, any recollection of previous visit to formal health services, and school attendance. Parents were not available for information. Permission to do screening was obtained by the school parents´ committee. School attendance and vaccine records were inspected but were too incomplete to yield information more meaningful than student oral report. Children who met criteria for referral received written information to give to their parents.

The criteria for malnourishment was based on WHO´s Growth reference charts and definitions, assessed by BMI-for-age (5–19 years) and z-score [15,16]. Children who met criteria (Table 1) were referred for further clinical assessment at the outpatient department (OPD) at EAUH. All children were given one deworming dose (Albendazole) to reduce the prevalence of helminth infections [17], according to WHO recommendations [18].

**Table 1.** Frequency of conditions resulting in referral of children screened.

Criteria for referral	Frequency (%) of referred children
Malnourished children with a body-mass-index (BMI) at least 3SD below the mean	115 (47.1)*
Vision impaired children (visual acuity < 0.5 at Snellen’s eye chart)	62 (25.4)**
Symptoms and/or signs of infection (e.g. skin, eye, ear, throat, UTI)	48 (19.7)
Symptoms of tuberculosis (weightloss, cough, haemoptysis, nightsweat, fever, chest pain, fatigue)	2 (0.8)
Any other abnormal finding	17 (7.0)***
Total	244 (100)

*Referrals based on direct readouts, by students, from WHO reference charts of BMI-for-age with z-scores. **Borderline cases were also referred ***Abdominal pain, headache, pale conjunctiva, enlarged lymph node, high blood pressure.

BMI-for-age with z-scores were calculated using the WHO AnthroPlus calculator [19]. A Chi square-test and group trend-test for nonparametric data was utilized to examine associations. Data were entered into Stata version 15.0.

## Results

We screened 2,093 school children, representing 90.2% of all the registered pupils; age ranged from 4 to 19 years (median age 12.0 y). The characteristics of the children are listed in Table 2. More boys (58.2%) than girls were present for this school-based screening; more boys (11.9%) were severely malnourished (−3SD) than girls (6.1%; chi-square p-value < 0.001); more girls (32%) reported school absenteeism vs boys (25.8%; p = 0.002). School absenteeism was less likely in vaccinated children (26.8%) vs unvaccinated (32.8%; p = 0.01).

**Table 2.** Characteristics of pupils screened at two primary schools in Hargeisa, 2017.

Attribute		Frequency (%)
	Total participants	2093 (100)
Gender	Boys	1218 (58.2)
	Girls	875 (41.8)
	Missing information	0
Age groups	4–7	177 (8.4)
	8–11	824 (39.4)
	12–15	952 (45.5)
	16–19	140 (6.7)
	Missing information	0
Grades	1–4	924 (44.2)
	5–8	1166 (55.8)
	Missing information	3
Orphan status	Both parents alive	1921 (92.6)
	One parent dead	150 (7.2)
	Both parents dead	3 (0.1)
	Missing information	19
Nutritional status (BMI-for-age)*	Severely malnourished (Z-score < −3)	198 (9.5)
	Moderately malnourished (Z-score between −2 and −3)	324 (15.5)
	Not malnourished (Z-score −1 ≤)	1569 (75.0)
	Missing information	2
Vaccination status	Any vaccination received	1410 (67.3)
	Not vaccinated	531 (25.4)
	Don’t know	152 (7.3)
	Missing information	0
Visual acuity both eyes	< 0.5	38 (1.8)
	0.5–0.7	92 (4.4)
	0.8 ≤	1955 (93.8)
	Missing information	8
Dental status	Dental caries (obvious cavitation)	541 (26.0)
	No dental caries	1538 (74.0)
	Missing information	14
Absenteeism	Absent from school last month due to sickness	594 (28.4)
	No absent	1498 (71.6)
	Missing information	1
Visits health services	First time visiting health professionals, health clinic or hospital	971 (46.5)
	Previous visiting health professionals, health clinic or hospital	1116 (53.5)
	Missing information	6

*Calculated using the WHO AnthroPlus calculator

The older children (aged 12–15 and 16–19) were more likely to be malnourished than the younger children (p < 0.001). Associations between nutrition status and other variables are shown in Table 3. The visual acuity did not differ by nutritional status (p = 0.31) or age groups (p = 0.13).

**Table 3.** Associations nutrition status and other variables.

Variables		Normal N (%)	BMI = −2 to −3 N (%)	BMI Z<-3 N (%)	Chi-square, p-value	Group trend-test, p-value	Missing
Age group	4–7	155 (87.6)	17 (9.6)	5 (2.8)	< 0.001	< 0.001	2
	8–11	660 (80.3)	109 (13.3)	53 (6.4)			
	12–15	665 (69.8)	172 (18.1)	115 (12.1)			
	16–19	89 (63.6)	26 (18.6)	25 (17.8)			
Gender	Boys	825 (68.4)	238 (19.7)	144 (11.9)	< 0.001	< 0.001	2
	Girls	744 (84.2)	86 (9.7)	54 (6.1)	(0.000)	(0.000)	
Visual acuity	< 0.5	25 (65.8)	7 (18.4)	6 (15.8)	0.31	0.13	10
	0.5 ≤	1539 (75.3)	316 (15.4)	190 (9.3)			
Orphan	Both parents alive	1448 (75.5)	290 (15.1)	181 (9.4)	0.31	0.19	21
	One or both parents dead	107 (69.9)	29 (19.0)	17 (11.1)			
Absenteeism	Yes	446 (75.1)	96 (16.2)	52 (8.7)	0.70	0.74	3
	No	1123 (75.1)	227 (15.2)	146 (9.7)			
Caries	Yes	413 (76.4)	64 (11.8)	64 (11.8)	0.004	0.70	16
	No	1143 (74.4)	260 (16.9)	133 (8.7)			
Vaccination	None	388 (73.1)	82 (15.4)	61 (11.5)	0.16	0.11	154*
	One or more	1064 (75.5)	223 (15.8)	122 (8.7)			

Footnotes:

*Includes 152 reporting ‘don’t know’.

Of the students screened 11.7% (N = 244) met referral criteria (Table 1). Only about half (6.0%, N = 126) came for consultation. Thirty-five (27.8%) had normal assessments. Primary diagnosis is reported in Table 4.

**Table 4.** Clinical diagnosis of children who presented for evaluation after receiving a referral.

Diagnosis (Criteria)	Frequency (%)
Severe malnourishment (BMI, Z-score −3)*	28 (22.2)
Impaired vision (Paediatric Snellen Chart Score < 0.5)**	27 (21.4)
Ear problems***	15 (11.9)
Trachoma (WHO simplified grading system of trachoma)	6 (4.8)
Allergic conjunctivitis	5 (4.0)
Other ailments****	10 (7.9)
Normal assessments	35 (27.8)
Total children referred who came for consultation at OPD	126 (100)

* All were given vitamin supplements, general nutritional advices and invitation to nutritional workshop. Haemoglobin blood test (Hb) was taken of the 20 most malnourished pupils. Only one had Hb lower than 12 g/100ml.

**All were referred to an optician.

***Ear problems: (acute otitis media, otitis externa, long-standing perforated tympani, ear wax).

****Other ailments: Urinary tract infection, bacterial tonsillitis, skin infection, persistent headache, heartburn with positive *Helicobacter Pylori* serology test, long persisting neck lump.

## Discussion

This study of children attending selected schools in Hargeisa successfully exposed trainees to a school-based health program and screened more than 2,000 children for treatable conditions which have the potential to impact their ability to learn.

We found a substantial number of children had conditions that are known to impact their ability to learn. Malnutrition rates were substantial and 25.0% of the children were malnourished. This is a higher prevalence compared to other surveys using BMI-for-age among primary and secondary school children in Nigeria (20%), Vietnam (11%) and India (22%) [20–22]. The famine and cholera outbreak in the study area in 2017 may have contributed to poor nutrition [23].

Our study showed that 6.2% of the children had visual acuity below 0.7, and 1.8% below 0.5. This is a lower prevalence compared to a survey conducted among 6056 Indian older school children, with a prevalence of 4.6% below 0.5 vision acuity [24]. A study from South Africa showed a prevalence of visual acuity below 0.66 at 16% among school children aged 14–20 years. They found an association between diet of low proteins, high carbohydrate and negligible fruit and vegetable intake and poor visual acuity [25]. In our study impaired vision was not significantly associated with malnutrition. Even though the incidence of myopia increases during and after puberty, we found no difference in visual acuity by age [26].

In our study children who reported evidence of any vaccination had less absenteeism. This might be an indicator of the parents’ behaviour benefitting the child health in general.

Dental caries, while not directly impacting learning, was an obvious problem affecting 26% of students.

Only 50% of the referred children showed up for further clinical assessments. Potential reasons for the low rate of consultation include financial costs, scepticism towards the health system, and students losing the referral form.

This study has multiple limitations. Self-reported data by children may be less accurate than information from parents. Student nurses with limited experience did the screening, but after the training with the on-site supervision we are reasonably confident with the results. With less than 50% of the children in the community attending school [3], it is difficult to make any generalizations from this data. It is likely that poorer families have less access to education given costs involved and non-school children may have worse health status [27]. These data are from a convenience sample of school youth making it inappropriate to generalize these results to all children in Hargeisa. That said, it is to our knowledge the only data. It is highly likely that these conditions are ubiquitous, though the specific frequencies reported may be an underestimate of the scope of the problem.

## Conclusion

This survey shows that a significant number of Somaliland school children in Hargeisa were malnourished and unvaccinated; suffered reduced visual acuity, obvious dental caries or treatable infections. For most of these children, this was the first encounter with any health professionals. Public schools present an excellent opportunity for public health action in fragile countries like Somaliland. Incorporating such school-based screening into health professional training provides practical exposure to public health for students and could be an entry point for public health action in general.

## Paper context

The first school health program in post-civil war Somaliland has provided valuable data on the health condition of 2093 public primary school children in Hargeisa. A significant number of children were malnourished and unvaccinated; many had reduced visual acuity, obvious dental caries or treatable infections. For most of these children, this was the first encounter with any health professionals. Therefore, a provision of simple routine primary health care to primary schools is needed.
